# Using serological diagnostics to characterize remaining high-incidence pockets of malaria in forest-fringe Cambodia

**DOI:** 10.1186/s12936-024-04859-5

**Published:** 2024-02-15

**Authors:** Mathilde Grimée, Costanza Tacoli, Mirco Sandfort, Thomas Obadia, Aimee R. Taylor, Amélie Vantaux, Leanne J. Robinson, Dysoley Lek, Rhea J. Longley, Ivo Mueller, Jean Popovici, Michael T. White, Benoît Witkowski

**Affiliations:** 1 Infectious Disease Epidemiology and Analytics, Institut Pasteur, Université Paris Cité, Paris, France; 2 Collège Doctoral, Sorbonne Université, Paris, France; 3 Malaria Research Unit, Institut Pasteur du Cambodge, Phnom Penh, Cambodia; 4 Malaria Parasites and Hosts, Institut Pasteur, Université Paris Cité, Paris, France; 5 Bioinformatics and Biostatistics Hub, Institut Pasteur, Université Paris Cité, Paris, France; 6 Burnet Institute, Melbourne, Australia; 7 Papua New Guinea Institute of Medical Research, Madang, Papua New Guinea; 8 Population Health and Immunity, The Walter and Eliza Hall Institute of Medical Research, Parkville, Australia; 9 National Centre for Parasitology, Entomology and Malaria Control, Phnom Penh, Cambodia; 10 School of Public Health, National Institute of Public Health, Phnom Penh, Cambodia; 11 Department of Medical Biology, The University of Melbourne, Parkville, Australia

**Keywords:** *Plasmodium vivax*, Serology, Risk stratification, Forest, Cambodia, Greater Mekong Subregion

## Abstract

**Background:**

Over the last decades, the number of malaria cases has drastically reduced in Cambodia. As the overall prevalence of malaria in Cambodia declines, residual malaria transmission becomes increasingly fragmented over smaller remote regions. The aim of this study was to get an insight into the burden and epidemiological parameters of *Plasmodium* infections on the forest-fringe of Cambodia.

**Methods:**

950 participants were recruited in the province of Mondulkiri in Cambodia and followed up from 2018 to 2020. Whole-blood samples were processed for *Plasmodium* spp*.* identification by PCR as well as for a serological immunoassay. A risk factor analysis was conducted for *Plasmodium vivax* PCR-detected infections throughout the study, and for *P. vivax* seropositivity at baseline. To evaluate the predictive effect of seropositivity at baseline on subsequent PCR-positivity, an analysis of *P. vivax* infection-free survival time stratified by serological status at baseline was performed.

**Results:**

Living inside the forest significantly increased the odds of *P. vivax* PCR-positivity by a factor of 18.3 (95% C.I. 7.7–43.5). Being a male adult was also a significant predictor of PCR-positivity. Similar risk profiles were identified for *P. vivax* seropositivity. The survival analysis showed that serological status at baseline significantly correlated with subsequent infection. Serology is most informative outside of the forest, where 94.0% (95% C.I. 90.7–97.4%) of seronegative individuals survived infection-free, compared to 32.4% (95% C.I.: 22.6–46.6%) of seropositive individuals.

**Conclusion:**

This study justifies the need for serological diagnostic assays to target interventions in this region, particularly in demographic groups where a lot of risk heterogeneity persists, such as outside of the forest.

**Supplementary Information:**

The online version contains supplementary material available at 10.1186/s12936-024-04859-5.

## Background

Through the intensification of global malaria control efforts, significant progress has been made worldwide towards its elimination over the past decades. Most endemic countries of the Asia–Pacific region and the Americas have set the goal of malaria elimination by 2030 [1, 2]. However, in recent years progress has stalled in many countries. Outside of Africa, as endemicity declines, an ever-increasing proportion of malaria cases are caused by *Plasmodium vivax* as opposed to the historically predominant *Plasmodium falciparum*. *Plasmodium vivax* is the most geographically widespread *Plasmodium* parasite, causing substantial morbidity with an estimated 14.3 million clinical malaria cases per year [3], and it is now the primary cause of malaria outside of Africa [4].

*Plasmodium vivax* is an understudied parasite causing low-density, often asymptomatic, infections. Nonetheless it causes significant morbidity. Several biological characteristics enable it to evade routine malaria control strategies, mostly targeted at *P. falciparum*. It has the particularity that it remains dormant in the liver, where it escapes acute blood-stage treatment and causes subsequent relapses. These dormant parasites, called hypnozoites, are virtually undetectable, making relapses impossible to disentangle unambiguously from reinfections. These particularities have contributed to maintaining the parasite reservoir and sustaining onward transmission [5].

The changing landscape of malaria in Cambodia is characteristic of the Greater Mekong Subregion [6, 7]. Like its neighbouring countries, it has set the ambitious goal of malaria elimination before 2030 [7] and deployed “last mile” accelerating strategies to achieve this target [8, 9]. Specifically, Cambodia projects *P. falciparum* malaria elimination by 2023 and all species human-malaria elimination by 2025 [10]. In Thailand, Vietnam and Cambodia, a steady decrease of reported malaria cases, as well as malaria mortality has been observed over the past decade. While the decline of *P. falciparum* has been continuous, dropping to less than 400 yearly cases in 2022, *P. vivax* has been shown to be more resilient to malaria-control efforts and decreasing more slowly [6, 11]. *Plasmodium* *vivax* has taken over as the primary parasite, accounting for at least three quarters of the malaria burden in Cambodia [7, 12, 13]. Most strategies set by the National Malaria Control Programme target blood-stage parasites [14], hence the marked difference in effectiveness towards *P. vivax* elimination. However, radical cure, which targets the liver-stage of the parasite as well as blood stages, has been deployed as part of a national *P. vivax* programme since 2021 [15].

As the overall prevalence of malaria in Cambodia declines, the heterogeneity in residual malaria transmission increases [10, 13, 16]. The national malaria burden becomes increasingly fragmented over smaller and more remote regions, such as in the high-incidence provinces Ratanakiri and Mondulkiri in the East of the country [7, 12]. The local economic activities in these provinces are primarily logging and agriculture, activities which are associated with increased malaria risk [13]. Being a vector-borne disease, malaria is highly environment-dependent. The main local vectors, primarily *Anopheles dirus* and *Anopheles minimus* are usually found in and around the forest making this the primary transmission site from mosquitoes to humans [17]. The remaining high-risk areas need to be identified and characterized to allow for the continued tailored anti-malaria efforts [6].

The aim of this study was to get an insight into the shifting burden of malaria in the forest-fringe of Cambodia using molecular diagnostics and novel serological exposure markers. This study helps support locally adapted, and environment-specific decision-making, as opposed to potentially less cost-effective, one-size-fits-all approaches, with the aim of malaria elimination in Cambodia [18–20].

## Methods

### Study design

Participants aged 2–80 who had resided in the study area for at least 3 months were recruited from 10 villages in the Kaev Seima district in the high-incidence province of Mondulkiri in Cambodia. Smaller villages were oversampled as described in [13]. The recruited individuals were observed for a baseline visit between December 2018 and January 2019, 12 visits at approximately monthly intervals between January 2019 and March 2020, and an additional three visits at two- to three-month intervals between June and December 2020 (Additional file 1: Fig. S1).

Data was collected on participants’ village of residence, household composition, age, and sex. On each visit, the participants’ haemoglobin levels, rapid diagnostic test results, *Plasmodium* spp. PCR testing, and species-specific *Plasmodium* PCR testing, if the genus-specific PCR was positive, were collected. Serological information was processed for the baseline visit only.

### Laboratory methods

#### PCR

The PCR laboratory methods were as described by Sandfort et al*.* [13]. The blood samples collected at the interview site were stored at 4 °C. They were then separated into plasma and cell pellet and frozen at − 20 °C. At Institut Pasteur of Cambodia in Phnom Penh, cell pellets were stored at − 20 °C and plasma at − 80 °C. Infections with *Plasmodium spp.* parasites were determined by real-time PCR. Then qPCR specific for *P. falciparum*, *P. vivax*, *Plasmodium malariae*, and *Plasmodium ovale* was performed.

#### Serology

IgG antibody responses to 14 proteins of *P. vivax* were measured using a Luminex platform, as described elsewhere [21, 22]. The panel of serological exposure markers (SEM) to *P. vivax* was previously validated [21] and consisted of 8 proteins: PVX_099980 (19 kDa C-terminal region of merozoite surface protein 1, PvMSP1-19), PVX_096995 and PVX_112670 (2 constructs of the tryptophan-rich antigen, Pv-fam-a), PVX_097625 (merozoite surface protein 8, putative, PvMSP8), PVX_097720 (merozoite surface protein 3, PvMSP3a), PVX_087885 (rhoptry-associated membrane antigen, putative, PvRAMA), PVX_094255 (reticulocyte binding protein 2b, N-terminal fragment, PvRBP2b) and KMZ83376.1d (erythrocyte-binding protein, PvEBP). To account for inter-plate variation, a standard curve was prepared using a plasma pool from hyper-immune adults from Papua New Guinea. Relative antibody units (RAU) or dilutions were obtained extrapolating the median fluorescence intensity (MFI) in a standard curve by a 5-parameter logistic model.

### Distribution of demographics factors

The cohort was described in terms of sex (“male”, “female”), age (“ ≤ 16 years”, or “children”, and “ > 16” years, or “adults”) and forest coverage of village of residence (“Inside forest”, “Forest fringe”, and “Outside forest”). The villages were classified in terms of their forest coverage based on a land cover analysis that was done in [17]. Villages where ≥ 50% of households had ≥ 10% forest cover in their vicinity as of 2018 were considered “Inside forest”. Those with ≥ 30% of households having ≥ 5% forest cover in their vicinity were classified as “Forest fringe”, and, finally, “Outside forest” otherwise. The rationale behind the definition of these risk factor variables comes from [13].

### Time-trends of *Plasmodium* spp. PCR-prevalence stratified by major demographic factors

For each follow-up month, from baseline to the last extra visit, the prevalence of PCR-detected infections with any *Plasmodium* species was computed. The prevalence was first computed for all study participants, then stratified by sex, then by age, then by forest coverage. It was finally stratified three ways, by sex, age, and forest coverage. To visualize the dominance of some *Plasmodium* species over others, the prevalence time trend was stratified by *Plasmodium* species. All these time trends were presented as line plots, corresponding to the time-dependent point estimates of prevalence, with a shaded area corresponding to the 95% binomial confidence interval.

### *Plasmodium vivax* PCR- and seropositivity at baseline

Due to the significant dominance of *P. vivax* over other *Plasmodium* species (Additional file 1: Figure S2, Table S1), all subsequent statistical modelling was performed on *P. vivax* PCR and seropositivity data exclusively. Individual serological information was available from the baseline visit in the form of continuous quantifications of antibody levels to a panel of *P. vivax* proteins. Using this data, a random forest classification of the study population into seropositive and seronegative as described in [21] was performed. This classification algorithm uses this antibody panel to estimate with 80% specificity and 80% sensitivity whether individuals were infected with *P. vivax* within the past nine months. The point-estimates of the prevalence of PCR-detected *P. vivax* infections, and the *P. vivax* seroprevalence in the cohort at baseline were calculated.

### Risk factors for* P. vivax* PCR- and seropositivity

To evaluate the demographic factors associated with *P. vivax* PCR-positivity, a mixed-effects logistic regression model was fitted, including a linear time-trend, all demographic predictors, and pairwise and three-way interactions between the three demographic predictors. The model above included an individual-based random intercept to account for the longitudinal structure of the data. The variance of the random intercept was reported as well as the predictor estimates, standard errors, and p-values. This model was then narrowed down to include only informative risk factors in the final model. Given that it is impossible to disentangle reinfections from relapses or continued infections in the case of PCR-detected infections on consecutive months within an individual, sequential positive results per individual were not treated differently at this stage. Each infection is treated as a novel infection. For the risk factor analyses, the three extra visits were excluded from the longitudinal analyses. These visits took place during the first wave of the COVID-19 pandemic in spring 2020, as social distancing measures and restrictions to forest logging activities were put in place in the study area [9].

Then, to investigate the association between age, sex, and forest coverage with *P. vivax* seropositivity at baseline, a multivariate logistic regression model was fitted. The three demographic predictors, and the significant pairwise interactions were included.

### Survival analysis of *P. vivax* infection-free survival time

To evaluate the predictive effect of seropositivity at baseline on subsequent PCR-positivity, an analysis of *P. vivax* infection-free survival time after baseline, stratified by serological status at baseline was performed. Kaplan-Meyer curves of infection-free survival over the study period, stratified by serological status at baseline were first drawn. Then the analysis was repeated only considering the subjects in the demographic groups found to be most at risk, and drew Kaplan-Meyer curves, stratified again by seropositivity at baseline. This analysis was used to determine whether seropositivity status was informative in isolating risk profiles beyond demographic groups. A Cox-proportional hazards model was computed for *P. vivax* infection, with seropositivity as a main predictor, adjusted by the demographic variables sex, age, and forest coverage. Significance of the association between seropositivity at baseline and the hazards of subsequent *P. vivax* infection was evaluated at significance level $\alpha$ = 0.05.

### Subgroup analysis of the performance of serodiagnosis for detecting future infections

Finally, it was evaluated in which subgroups of the cohort seropositivity at baseline would be predictive for a future infection in the time frame of the cohort was performed. True positives (TP) were defined as individuals who were seropositive at baseline who have at least one PCR-detected infection over the study period, and true negatives (TN) as individuals who were seronegative at baseline and did not develop a PCR-detected infection throughout the study period. In each subgroup the positive predictive value (PPV) was presented, i.e., the proportion of individuals who were seropositive at baseline who did develop an infection (TP/all seropositives), and the negative predictive value (NPV), i.e., the proportion of individuals who were seronegative at baseline who did not develop an infection during the study period (TN/all seronegatives). The corresponding 95% binomial confidence intervals were presented. This analysis allows the exploration of how well the classification algorithm developed in [21] which establishes seropositivity based on probability of past infection, is also useful in predicting the risk of future infection in different subgroups.

### Software

All statistical and graphical analyses were performed in *R* 4.2.2 (2022–10-31).

## Results

### Study participants and observations

A total of 950 participants from 391 households were observed for an average of 10.45 of the 16 planned visits, with 285 complete cases, and 85 participants lost to follow-up immediately after baseline. A total of 9928 samples were collected. Serological information, in the form of continuous quantifications of antibody levels to a panel of *P. vivax* proteins, was available for 934 subjects at the baseline visit (Table 1).

**Table 1:** Cohort description

# Participants	950
# Samples for PCR	9928
# Samples for serology	934
Sample collection date	Dec 18-Dec 20
% Female	53.47%
% Male	46.53%
Age distribution	Range: 5 - 66 years
	Mean 22.68 years
	Median 19.5 years
% Children	42.21%
% Adults	57.79%
% living inside forest	24.95%
% living on forest fringe	34.84%
% living outside forest	40.21%
Laboratory data	*Plasmodium* spp. PCR
	*Plasmodium*-specific PCR
	Antibody titres to
	14 *P.vivax* proteins

**Fig. 1:**
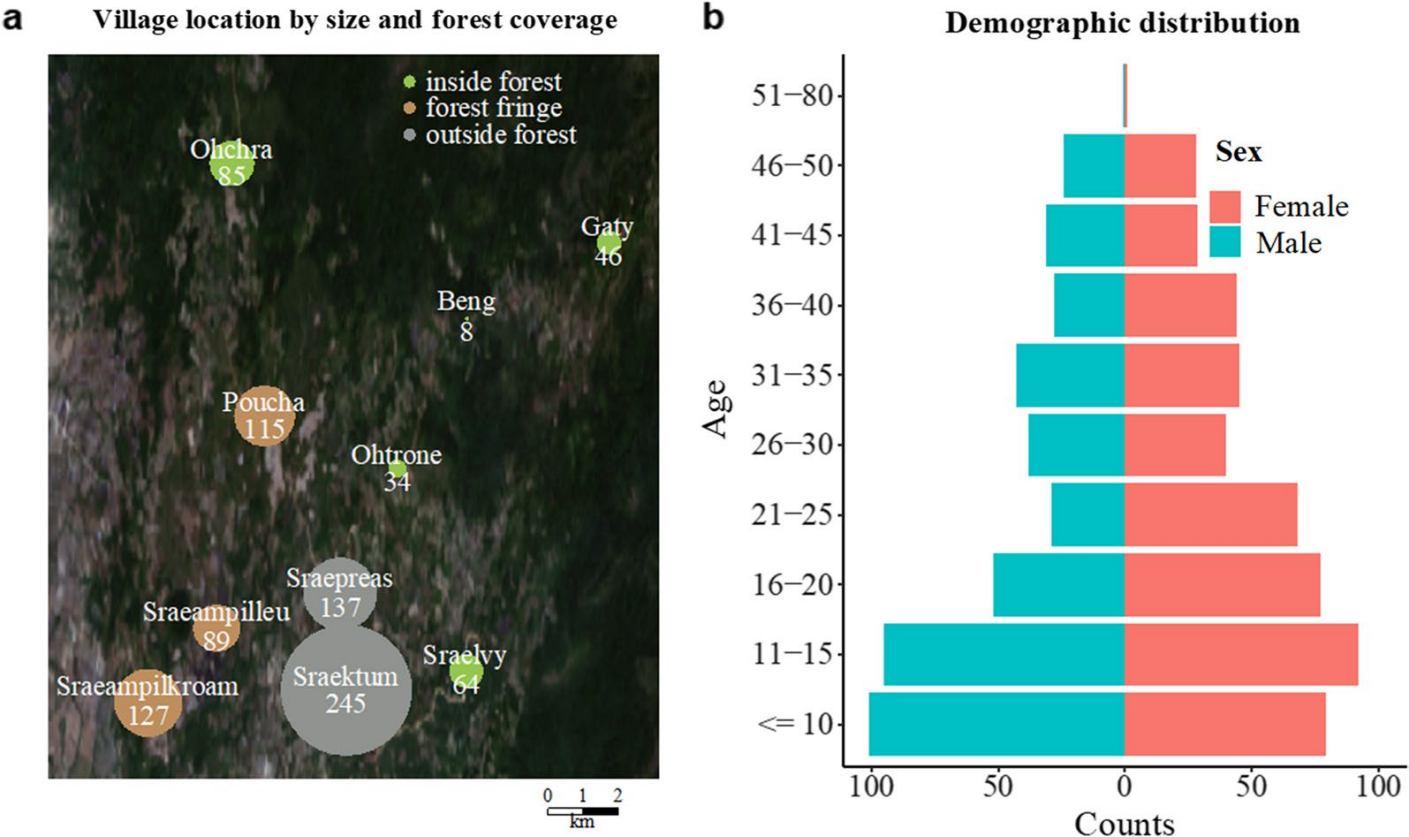
**a** Geographical distribution of villages and corresponding participant number. **b** Demographic pyramid, by age and gender

Figure 1b shows the distribution of age groups in the participants observed at baseline, stratified by sex. There was a slight overrepresentation of male participants in children until age 16, followed by an overrepresentation of female participants from age 16 onwards. In terms of forest coverage, participants living inside the forest, outside the forest, and on the forest fringe were approximately equally represented (Fig. 1a).

### Time-trends of *Plasmodium* spp. PCR-prevalence stratified by major demographic factors

**Fig. 2:**
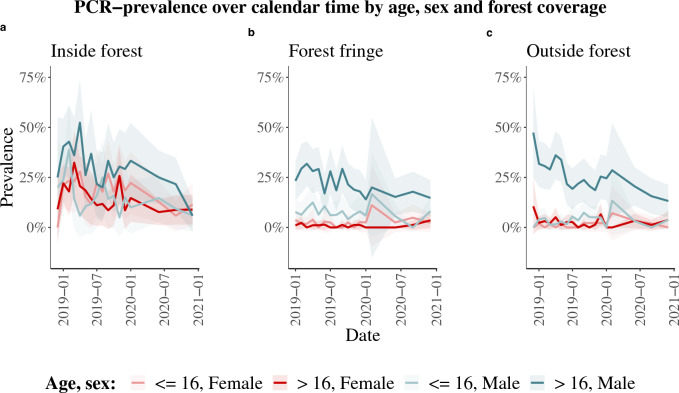
*Plasmodium* spp. PCR-prevalence over study period prevalence (lines) with 95% binomial confidence interval (shaded area) by sex and age **a** inside the forest, **b** on the forest fringe, and **c** outside the forest

Over the study period, the prevalence of PCR-detected *Plasmodium* infections significantly decreased by almost half across the study population, from 14% (11–16%) to 7% (5–9%) (Additional file 1: Fig. S3a). The time trends of *Plasmodium* infection prevalence were visualized by a three-way stratification – by sex, age, and forest coverage. Figure 2 shows a clear trend, that the further away from the forest, the more male adults separate from the rest of the population in terms of *P. vivax* prevalence.

### Distribution of *P. vivax* PCR- and seropositivity at baseline

The distribution of *P. vivax* PCR-positivity at baseline is presented in Table 2.

**Table 2:** Distribution of *P. vivax* infection

Forest coverage	PCR positivity	Sex	PCR positivity	Age	PCR positivity
**a.** PCR-prevalence					
Outside forest	0.10	Female	0.02	<= 16	0.01 n=73
	n=382		n=197	> 16	0.02 n=124
		Male	0.19	<= 16	0.06 n=90
			n=185	> 16	0.32 n=95
Forest fringe	0.08	Female	0.02	<= 16	0.03 n=63
	n=331		n=171	> 16	0.01 n=108
		Male	0.14	<= 16	0.08 n=78
			n=160	> 16	0.20 n=82
Inside forest	0.23	Female	0.19	<= 16	0.17 n=54
	n=237		n=140	> 16	0.20 n=86
		Male	0.30	<= 16	0.23 n=43
			n=97	> 16	0.35 n=54

Forest coverage	Seropositivity	Sex	Seropositivity	Age	Seropositivity
**b.** seroprevalence in the cohort at baseline					
Outside forest	0.23	Female	0.09	<= 16	0.08 n=72
	n=373		n=193	> 16	0.10 n=121
		Male	0.37	<= 16	0.13 n=87
			n=180	> 16	0.59 n=93
Forest fringe	0.26	Female	0.18	<= 16	0.11 n=63
	n=330		n=171	> 16	0.22 n=108
		Male	0.35	<= 16	0.14 n=78
			n=159	> 16	0.54 n=81
Inside forest	0.53	Female	0.47	<= 16	0.33 n=52
	n=231		n=137	> 16	0.56 n=85
		Male	0.62	<= 16	0.40 n=42
			n=94	> 16	0.79 n=52

Of the 950 cohort subjects, 119 were PCR positive at baseline. The lowest prevalence was found in female participants outside the forest (1% in children and 2% in adults), and on the forest fringe (3% in children and 1% in adults). The highest prevalence of *P. vivax* was found in male adults inside the forest at 35%, preceded by male adults outside the forest at 32%, then male children inside the forest at 23% (Table 2a).

Of the 934 subjects for which serological data was available, a total of 294 were found to be seropositive. The lowest seroprevalence was found in female children outside the forest at 8%, followed by female adults outside the forest at 10%, then female children on the forest fringe at 11%. The highest seroprevalence to *P. vivax* was found in male adults inside the forest at 79%, followed by male adults outside the forest at 59%, female adults inside the forest at 56%, and finally male adults on the forest fringe at 54% (Table 2b).

### Predictors of *P. vivax* PCR- and seropositivity

A mixed-effects logistic regression model including all demographic predictors as well as pairwise and three-way interactions between them was computed and is shown in Additional file 1: Table S2. The most parsimonious and informative model is shown in Table 3a. This model shows us that there is a significant downward time-trend in *P. vivax* prevalence. This trend is not significantly different in different demographic group as shown in Additional file 1: Tables S3-S5. Living inside the forest, compared to outside the forest, significantly increased the odds of *P. vivax* PCR-positivity by a factor of 18.3 (95% C.I. 7.7–43.5). Being a male adult was also a significant predictor of PCR-positivity.

**Table 3:** Parameter estimates for

	Estimate	Std. Error	p-value
**a.** a mixed-effects logistic regression of *P. vivax* PCR-detected infections			
(Intercept)	− 8.263	0.598	0.000
Time (centered)	− 0.002	0.000	0.000
Sex (Male)	1.026	0.549	0.062
Age (> 16)	− 0.192	0.543	0.723
Forest (inside forest)	2.909	0.441	0.000
Forest (forest fringe)	0.144	0.425	0.735
Sex (Male):Age (> 16)	3.251	0.717	0.000
Individual random intercept		4.044	

	Estimate	Std. Error	p-value
**b.** *Plasmodium vivax* seropositivity at baseline			
(Intercept)	− 2.391	0.248	0.000
Sex (Male)	0.365	0.281	0.193
Age (> 16)	0.735	0.249	0.003
Forest (inside forest)	1.702	0.205	0.000
Forest (forest fringe)	0.243	0.194	0.211
Sex (Male):Age (> 16)	1.374	0.342	0.000

The reference level is a female child outside the forest

A simple logistic regression on the seropositivity data at baseline, using the same predictors as above, is shown in Table 3b. This model indicates that male individuals, adults, and people living inside the forest are significantly at risk of being seropositive for *P. vivax*. Additionally, there is a significant interaction between being male and being an adult. More risk factors were identified for seropositivity, given the higher statistical power in detecting seropositivity over a larger time window, than PCR-infections at a specific time. Therefore, these findings are in line with the risk factors identified for *P. vivax* PCR-positivity.

### *Plasmodium vivax*-infection-free survival stratified by seropositivity at cross-section

**Fig. 3:**
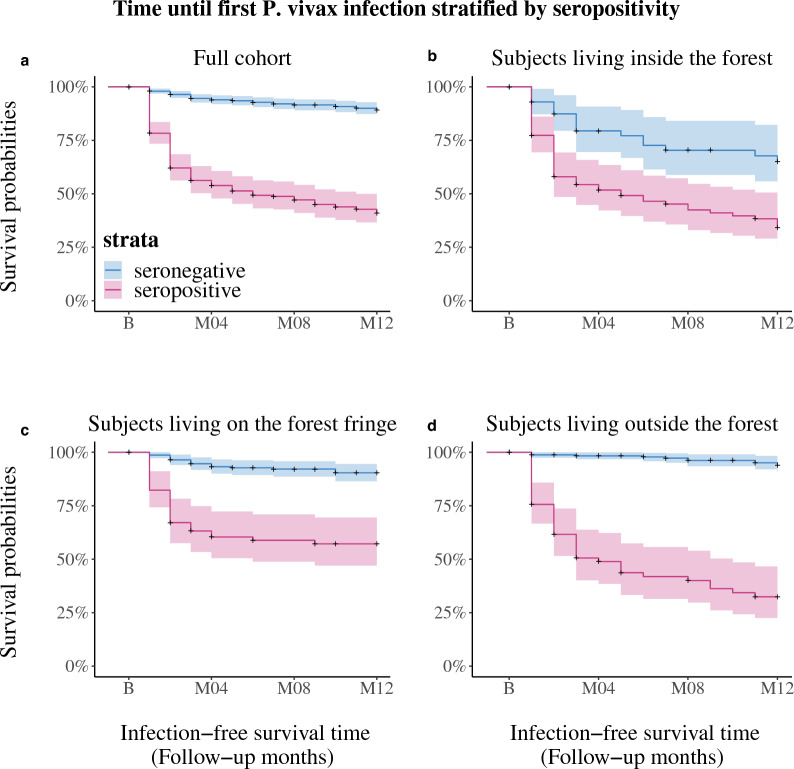
Kaplan-Meyer estimates of *P. vivax* infection-free survival (lines) with 95% confidence interval (shaded area) stratified by seropositivity status at the cross-section for **a** the whole cohort, **b** individuals inside the forest, **c** individuals living on the forest fringe, and **d.** individuals living outside the forest

To explore the relationship between seropositivity and subsequent risk of infection, a survival analysis of time until first infection after baseline was performed on the 934 subjects for which serological information was available. Figure 3a shows that globally, serology significantly affects subsequent infection, with 89.2% (95% C.I. 86.4–92.1%) infection-free survival in seronegative individuals, compared to 41.0% (95% C.I. 34.9–48.2%) in seropositive individuals. Figure 3b shows that inside the forest, 65.1% (95% C.I. 52.9–80.2%) of seronegative individuals survived infection-free, compared to 34.1% (95% C.I. 25.1–46.3%) of seropositive individuals. Figure 3c shows that on the forest fringe, 90.3% (95% C.I. 86.4–94.5%) of seronegative individuals survived infection-free, compared to 57.2% (95% C.I. 47.0–69.5%) of seropositive individuals. Finally, Fig. 3d shows that outside the forest, 94.0% (95% C.I. 90.7–97.4%) of seronegative individuals survived infection-free, compared to 32.4% (95% C.I. 22.6–46.6%) of seropositive individuals.

This survival analysis shows that serology is particularly informative outside of the forest and on the forest fringe. A table for the number of individuals at risk at every time point, stratified by seropositivity status can be found in Additional file 1: Table S8.

The Cox-proportional hazards models shown in Table 4b and Table 4c show that when considering individuals outside the forest and on the forest fringe, after adjusting for the demographic factors age and sex, shown before to be significantly associated with *P. vivax* infection, seropositivity is still significantly associated with higher hazards of new infections. Outside the forest, the hazard of new infection is exp(1.627) = 5.09 (95% C.I. 2.04–12.72) times higher for seropositive individuals compared to seronegative individuals, and on the forest fringe it is exp(1.055) = 2.87 (95% C.I. 1.35–6.13) times higher. Table 4a shows that seropositivity is not significantly associated with higher hazards of new infections when considering individuals inside the forest. These results confirm that serology is most informative in the settings outside the forest and on the forest fringe. As shown in Additional file 1: Table S9, the models did not violate the proportional hazards assumption.

**Table 4:** Cox-proportional hazards model of infection for individuals

Inside forest	Estimate	Std. Error	p-value
**a.** inside the forest			
Sex (Male)	− 0.297	0.558	0.595
Age (> 16 years)	− 0.287	0.483	0.552
Seropositive (yes)	0.149	0.378	0.694
Sex (Male):Age (> 16 years)	1.496	0.711	0.035

Forest fringe	Estimate	Std. Error	p-value
**b.** on the forest fringe			
Sex (Male)	2.074	1.054	0.049
Age (> 16 years)	0.301	1.157	0.795
Seropositive (yes)	1.055	0.379	0.005
Sex (Male):Age (> 16 years)	0.353	1.226	0.774

Outside forest	Estimate	Std. Error	p-value
**c.** outside the forest			
Sex (Male)	0.485	0.866	0.576
Age (> 16 years)	− 1.198	1.225	0.328
Seropositive (yes)	1.627	0.458	0.000
Sex (Male):Age (> 16 years)	2.607	1.357	0.055

### Comparative subgroup analysis of the performance of seropositivity for predicting future infections

In this analysis, the best PPV was found outside the forest *vs.* inside or on the forest fringe, in males *vs.* in female individuals, and in children *vs.* in adults. The best NPV was also found outside the forest *vs.* inside or on the forest fringe, but in female *vs.* in male individuals, and again in children *vs.* in adults (Table 5).

**Table 5:** Positive predictive value (PPV) and negative predictive value (NPV) of seropositivity at baseline for predicting infections during the study period

Group	PPV (%)	95%CI	NPV (%)	95%CI
Whole cohort	46.08	(42.88%, 49.27%)	92.20	(90.48%, 93.92%)
Inside forest	45.53	(39.11%, 51.95%)	83.33	(78.53%, 88.14%)
Forest fringe	38.37	(33.13%, 43.62%)	91.80	(88.84%, 94.76%)
Outside forest	54.76	(49.71%, 59.81%)	95.85	(93.82%, 97.87%)
Males	58.66	(54.02%, 63.30%)	86.22	(82.97%, 89.47%)
Females	26.32	(22.46%, 30.17%)	96.12	(94.43%, 97.81%)
Adults	45.98	(41.78%, 50.19%)	90.82	(88.39%, 93.26%)
Children	46.38	(41.45%, 51.30%)	93.54	(91.11%, 95.97%)
Male adults	60.71	(54.35%, 67.08%)	75.58	(69.98%, 81.18%)

The PPV is defined as the proportion of all seropositive individuals at baseline who go on to have a PCR-detected infection during the study period. The NPV is defined as the proportion of all seronegative individuals at baseline who go on to remain infection-free for the whole study duration

Outside the forest, 54.76% (95% C.I. 48.71–59.81%) of seropositive individuals developed an infection and 95.85% (95% C.I. 93.82–97.87%) of seronegative individuals remained infection free over the study period. This confirms that it is really outside the forest that using seropositivity is most helpful as a tool for predicting future infection (PPV) as well as infection-free survival (NPV).

## Discussion

Using longitudinal data of PCR-detected *P. vivax* infection, in the Mondulkiri province of Cambodia, this study was able to describe the main demographic risk factors associated with *P. vivax* infection, and the association of seropositivity with infection-free survival. Male adults and individuals living inside the forest are most at risk for *P. vivax* infections, which is in line with the extensive research done in the Greater Mekong Subregion [9, 13, 23–27]. Additionally, it was shown that seropositivity is a useful tool for risk identification and a proxy measure of exposure useful to disentangle unexplained variation in some demographic risk groups.

This study reflects the changing burden of malaria in South-East Asia and the Greater Mekong Subregion. Over past decades, significant malaria control has been achieved in Cambodia and its neighbouring countries [6, 7, 13]. Small-scale, geographically focused studies, provide important insights into the mechanisms of this elimination and provide evidence of specific epidemiological profiles and remaining pockets of risk [13, 25, 26, 28–32]. Understanding these risk profiles is crucial to effectively provide targeted efforts for the successful elimination of resilient malaria in the Greater Mekong Subregion [6]. The forest and forest-fringe in Cambodia is a rapidly evolving environment that reshapes the malaria risk landscape with it [17]. This study provides specific insights into this changing epidemiology of malaria on the forest fringe.

Over the study period, the prevalence of PCR-detected *Plasmodium* infections decreased by half, from 14% (95% C.I. 11–16%) to 7% (95% C.I. 5–9%). The 1010 infections detected by PCR were almost exclusively *P. vivax* infections (Additional file 1: Table S1). While *P. falciparum* has long been the predominant malaria species in this region, elimination efforts have been largely successful and yearly national case numbers have dropped significantly, making *P. vivax* the most common parasite in the region [7, 12, 13]. Thus, the epidemiological results of this study are in line with the current epidemic landscape in Cambodia and the Greater Mekong Subregion at large. It should be noted here that considering the COVID-19 pandemic, some external factors such as changes in social behaviour as well as restrictions to forest-related activities towards the end of the study period were potentially not accounted for in the analysis and could have contributed to the visible decline in *Plasmodium* infections [9]. However, according to the Mekong Malaria Elimination: epidemiology summaries 2019–2023, a general decline in cases and deaths in Cambodia continued past the 2020 COVID-19 pandemic [6].

The stratified demographic risk factor analysis, via mixed effects logistic regression, showed that the highest malaria prevalence was found in male adults, and in individuals living inside the forest. Moreover, the three-way stratification, by sex, age and forest coverage showed that male adults separate from all other demographic groups the further away from the forest they live.

This is characteristic of two different types of exposure within and outside of the forest: peri-domestic and occupational [33]. Inside the forest, all demographic groups exhibit roughly the same prevalence, indicating infections occurring around the household. Outside of the forest patterns characteristic of occupational exposure were observed, where mostly adult males are affected, being the demographic group mostly concerned with forest-related work activities such as logging [13]. On the forest fringe, a probable mixture of both types of exposure was observed. It is thus mostly male adults and people living inside the forest who are most at risk of exposure to *P. vivax* malaria. Through a logistic regression, similar risk profiles were identified for seropositivity at baseline as for PCR-positivity throughout the study.

A survival analysis showed that seropositivity at baseline was a determining factor for infection-free survival during the cohort for people living outside the forest and on the forest fringe. The most striking effect occurred in residents outside the forest and on the forest fringe. The effect was still significant after adjusting for age and sex. For residents of villages inside the forest, a difference in reinfection risk between seropositive and seronegative individuals was observed, but the effect was no longer significant after adjusting for age and sex. This is what could be expected from previous research done on serology as a diagnostic tool. It is precisely in low-prevalence settings, where much heterogeneity exists, that serology can help disentangle risk profiles [34, 35]. It must be noted that this result is a compound effect of relapses and reinfections. Indeed, the serological classification algorithm was specifically designed to detect individuals infected within the past 6–9 months [21]. Therefore, some of the shorter infection-free survival time in seropositive individuals must be due to relapses from recent primary infections. Given that relapses and reinfections are virtually impossible to disentangle unambiguously [36], it is impossible to quantify what share of the effect come from relapses and what share to the stability of risk profiles over time.

Finally, a comparative subgroup analysis of the performance of seropositivity for predicting future infections was performed. It could be seen that in terms of positive predictive value and negative predictive value, seropositivity worked best at predicting future infections outside the forest, in children and in female participants, i.e., subgroups with lower prevalence. All in all, these results show that serological information is most helpful in singling-out individuals most at-risk of infection and who would most benefit from public health interventions or prophylactic treatment in settings with low background prevalence, such as outside the forest.

A few limitations caveat the conclusions that can be drawn from these results. First, due to the impossibility to disentangle relapses from reinfections, every PCR-confirmed case was treated as a novel infection in the longitudinal analyses. However, in this setting, a high proportion of *P. vivax* cases are relapses, which could artifactually strengthen the clustering effect. Furthermore, only one cross-sectional serological measurement was used in the analysis rather than longitudinal measurements. It remains to be studied how much information would be gained by utilizing more frequent serological data. Nonetheless, showing that baseline seropositivity measurements are already informative at explaining variations in exposure within demographic groups is encouraging for the use of serology as a risk-identification tool in future studies [37].

This study gives some evidence that in this region, malaria clusters in specific individuals consistently over time. This stability of risk profiles in contrast to spatially sporadic malaria, has the potential to facilitate targeted interventions. To take advantage of this however, it is necessary to properly characterize and identify the individuals who are persistently at-risk of infection. While demographic factors are a good first approach, serological markers can give a more refined indication of who these at-risk individuals are. It was shown here that serological information is a particularly valuable tool to absorb the unexplained remaining variance of risk present in demographic subgroups where a lot of heterogeneity persists. While it is unclear what share of this effect is due to relapses or to the persistence of risk profiles, this is of relatively little importance from a programmatic standpoint. In this region, the main targeted intervention being considered for the future is radical cure with primaquine or prophylactic treatment. It was shown here that using serological information adds significant value in predicting future infections, from relapses or reinfections, in low-prevalence settings, where, most likely, more risk heterogeneity persists. Future research could use mathematical modelling to study the impact of targeting these risk groups on the parasite reservoir and on ongoing transmission with a serological test-and-treat intervention.

## Conclusion

The results of this study show that *P. vivax* malaria is not a fleeting phenomenon in the Mondulkiri province. The stability of risk profiles on the medium-term is evident. It is the same small group of individuals that are consistently affected by malaria infections, male adults and people living inside the forest. While this is a pattern that has been observed at larger spatial resolutions [33], it still holds when zooming into finer pockets of high incidence. While partitioning the population according to main demographic factors can be a rough indication of risk groups (e.g., male adults and people living inside the forest), significant amounts of variation are still present within subgroups with lower background prevalence. In these settings, serological assessments are a robust and consistent way of singling out individuals in need of targeted treatment. Therefore, this study justifies the need for serological diagnostic assays to target interventions in this region.

### Supplementary Information

**Additional file 1: **Variable descriptions, additional analyses and model assumption tests

## Data Availability

The *R*-scripts used to perform the analyses will be made available on GitHub and the de-identified data will be uploaded on ClinEpiDB, at https://clinepidb.org/ce/app/. In the meantime, code and data are available from the corresponding author upon reasonable request.

## References

[1] Asia Pacific Leaders Malaria Alliance (2013). East Asia Summit adopts unprecedented regional malaria goal.

[2] Pan American Health Organization, WHO. Central America and Hispaniola seek to eliminate malaria by 2025. Geneva, World Health Organization, 2013.

[3] Battle KE, Lucas TC, Nguyen M, Howes RE, Nandi AK, Twohig KA (2019). Mapping the global endemicity and clinical burden of *Plasmodium vivax*, 2000–17: a spatial and temporal modelling study. Lancet.

[4] Robinson LJ, Wampfler R, Betuela I, Karl S, White MT, Li Wai Suen CS (2015). Strategies for understanding and reducing the *Plasmodium vivax* and *Plasmodium ovale* hypnozoite reservoir in Papua New Guinean children: a randomised placebo-controlled trial and mathematical model. PLoS Med.

[5] Mueller I, Galinski MR, Baird KJ, Carlton JM, Kochar DK, Alonso PL (2009). Key gaps in knowledge of *Plasmodium vivax*, a neglected human malaria parasite. Lancet Infect Dis.

[6] WHO. Mekong Malaria Elimination Programme: epidemiology summary. Geneva, World Health Organization, 2023.

[7] WHO. Mekong Malaria Elimination Programme: countries of the Greater Mekong zero in on falciparum malaria. Geneva, World Health Organization, 2019.

[8] WHO. Mekong Malaria Elimination Programme: countries of the Greater Mekong ready for the “last mile” of malaria elimination. Geneva, World Health Organization, 2020.

[9] WHO. Mekong Malaria Elimination Programme. Accelerating Malaria Elimination in the Greater Mekong. Geneva, World Health Organization, 2022.

[10] Ministry of Health, Kingdom of Cambodia. Cambodia Malaria elimination action framework 2021–2025. Cambodia, Phnom Penh, 2021.

[11] Price RN, Commons R, Battle K, Thriemer KE, Mendis K. *Plasmodium vivax* in the era of the shrinking *P. falciparum* map. Trends Parasitol*.* 2020;36:560–70. 10.1016/j.pt.2020.03.009 PMC7297627 32407682

[12] National Center for Parasitology, Entomology and Malaria Control (CNM). Annual Report 2018. Phnom Penh, 2019.

[13] Sandfort M, Vantaux A, Kim S, Obadia T, Pepey A, Gardais S (2020). Forest malaria in Cambodia: the occupational and spatial clustering of *Plasmodium vivax* and *Plasmodium falciparum* infection risk in a cross-sectional survey in Mondulkiri province. Cambodia Malar J.

[14] Ministry of Health, Kingdom of Cambodia. Cambodia Malaria Elimination Action Framework 2016–2020. Cambodia, Phnom Penh, 2016.

[15] President’s malaria initiative. Malaria Operational Plan FY 2023, Cambodia. Cambodia, Phnom Penh, 2023.

[16] Guyant P, Canavati SE, Chea N, Ly P, Whittaker MA, Roca-Feltrer A (2015). Malaria and the mobile and migrant population in Cambodia: a population movement framework to inform strategies for malaria control and elimination. Malar J.

[17] Pepey A, Souris M, Vantaux A, Morand S, Lek D, Mueller I (2020). Studying land cover changes in a malaria-endemic Cambodian District: considerations and constraints. Remote Sensing.

[18] Strategic Advisory Group on Malaria Eradication (2020). Malaria eradication: benefits, future scenarios and feasibility.

[19] Stresman G, Bousema T, Cook J (2019). Malaria hotspots: is there epidemiological evidence for fine-scale spatial targeting of interventions?. Trends Parasitol.

[20] Bannister-Tyrrell M, Krit M, Sluydts V, Tho S, Sokny M, Mean V (2019). Households or hotspots? Defining intervention targets for malaria elimination in Ratanakiri province. Eastern Cambodia J Infect Dis.

[21] Longley RJ, White MT, Takashima E, Brewster J, Morita M, Harbers M (2020). Development and validation of serological markers for detecting recent *Plasmodium vivax* infection. Nat Med.

[22] Mazhari R, Brewster J, Fong R, Bourke C, Liu ZSJ, Takashima E (2020). A comparison of non-magnetic and magnetic beads for measuring IgG antibodies against *Plasmodium vivax* antigens in a multiplexed bead-based assay using Luminex technology (Bio-Plex 200 or MAGPIX). PLoS ONE.

[23] Kunkel A, Nguon C, Iv S, Chhim S, Peov D, Kong P (2021). Choosing interventions to eliminate forest malaria: preliminary results of two operational research studies inside Cambodian forests. Malar J.

[24] Sovannaroth S, Ngor P, Khy V, Dunn JC, Burbach MK, Peng S (2022). Accelerating malaria elimination in Cambodia: an intensified approach for targeting at-risk populations. Malar J.

[25] Kerkhof K, Sluydts V, Heng S, Kim S, Pareyn M, Willen L (2016). Geographical patterns of malaria transmission based on serological markers for falciparum and vivax malaria in Ratanakiri. Cambodia Malar J.

[26] Tripura R, Peto TJ, Veugen CC, Nguon C, Davoeung C, James N (2017). Submicroscopic *Plasmodium* prevalence in relation to malaria incidence in 20 villages in western Cambodia. Malar J.

[27] Dysoley L, Kaneko A, Eto H, Mita T, Socheat D, Börkman A (2008). Changing patterns of forest malaria among the mobile adult male population in Chumkiri district. Cambodia Acta Trop.

[28] Durnez L, Pareyn M, Mean V, Kim S, Khim N, Menard D (2018). Identification and characterization of areas of high and low risk for asymptomatic malaria infections at sub-village level in Ratanakiri. Cambodia Malar J.

[29] Imwong M, Nguyen TN, Tripura R, Peto TJ, Lee SJ, Lwin KM (2015). The epidemiology of subclinical malaria infections in South-East Asia: findings from cross-sectional surveys in Thailand-Myanmar border areas, Cambodia, and Vietnam. Malar J.

[30] Parker DM, Tripura R, Peto TJ, Maude RJ, Nguon C, Chalk J (2017). A multi-level spatial analysis of clinical malaria and subclinical Plasmodium infections in Pailin province. Cambodia Heliyon.

[31] Tripura R, Peto TJ, Chalk J, Lee SJ, Sirithiranont P, Nguon C (2016). Persistent *Plasmodium falciparum* and *Plasmodium vivax* infections in a western Cambodian population: implications for prevention, treatment and elimination strategies. Malar J.

[32] Bosman P, Stassijns J, Nackers F, Canier L, Kim N, Khim S (2014). *Plasmodium* prevalence and artemisinin-resistant falciparum malaria in Preah Vihear province, Cambodia: a cross-sectional population-based study. Malar J.

[33] Lana R, Nekkab N, Siqueira AM, Peterka C, Marchesini P, Lacerda M (2021). The top 1%: quantifying the unequal distribution of malaria in Brazil. Malar J.

[34] Rosado J, White MT, Longley RJ, Lacerda M, Monteiro W, Brewster J (2021). Heterogeneity in response to serological exposure markers of recent *Plasmodium vivax* infections in contrasting epidemiological contexts. PLoS Negl Trop Dis.

[35] Chotirat S, Nekkab N, Kumpitak C, Hietanen J, White MT, Kiattibutr K (2021). Application of 23 novel serological markers for identifying recent exposure to *Plasmodium vivax* parasites in an endemic population of Western Thailand. Front Microbiol.

[36] Taylor AR, Watson JA, Chu CS, Puaprasert K, Duanguppama J, Day NPJ (2019). Resolving the cause of recurrent *Plasmodium vivax* malaria probabilistically. Nat Commun.

[37] Kartal L, Mueller I, Longley RJ (2023). Using serological markers for the surveillance of *Plasmodium vivax* malaria: a scoping review. Pathogens.

