# Supplementary material for: Using serological diagnostics to characterize remaining high-incidence pockets of malaria in forest-fringe Cambodia
Source: Malar J. 2024 Feb 15;23:49. doi: 10.1186/s12936-024-04859-5 (PMC10870639; doi:10.1186/s12936-024-04859-5)
Supplement: Supplementary file 1 — Additional file 1: Variable descriptions, additional analyses and model assumption tests [file 12936_2024_4859_MOESM1_ESM.pdf]

Using serological diagnostics to characterize remaining high-incidence pockets of malaria in forest-fringe Cambodia

Supplementary materials

Mathilde Grimée[1,2*], Costanza Taccoli[3], Mirco Sandfort[2,4], Thomas Obadia[1,5], Aimee R. Taylor[1], Amélie Vantaux[3,6], Leanne J. Robinson[7,8,9], Dysoley Lek[10,11], Rhea J. Longley[9,12], Ivo Mueller[4,9,10], Jean Popovici[3], Michael T. White[1,4*†], Benoît Witkowski[3,6*†]

[1]Infectious Disease Epidemiology and Analytics, Institut Pasteur, Université Paris Cité, Paris, France

[2]Sorbonne Université, Collège doctoral, Paris, France

[3]Malaria Research Unit, Institut Pasteur du Cambodge, Phnom Penh Cambodia.

[4]Malaria Parasites and Hosts, Institut Pasteur, Université Paris Cité, Paris, France

[5]Bioinformatics and Biostatistics Hub, Institut Pasteur, Université Paris Cité, Paris, France

[6]Genetic and Biology of Plasmodium, Institut Pasteur de Madagascar, Antananarivo, Madagascar

[7]Burnet Institute, Melbourne, Australia

[8]Papua New Guinea Institute of Medical Research, Madang, Papua New Guinea.

[9]Population Health and Immunity, The Walter and Eliza Hall Institute of Medical Research, Parkville, Australia

[10]National Centre for Parasitology, Entomology and Malaria Control, Phnom Penh, Cambodia

[11]School of Public Health, National Institute of Public Health, Phnom Penh, Cambodia

[12]Department of Medical Biology, The University of Melbourne, Parkville, Australia

*Correspondence: mathilde.grimee@pasteur.fr, michael.white@pasteur.fr or bwitkowski@pasteur.mg

†Michael White and Benoît Witkowski contributed equally to the work.

1. Study design

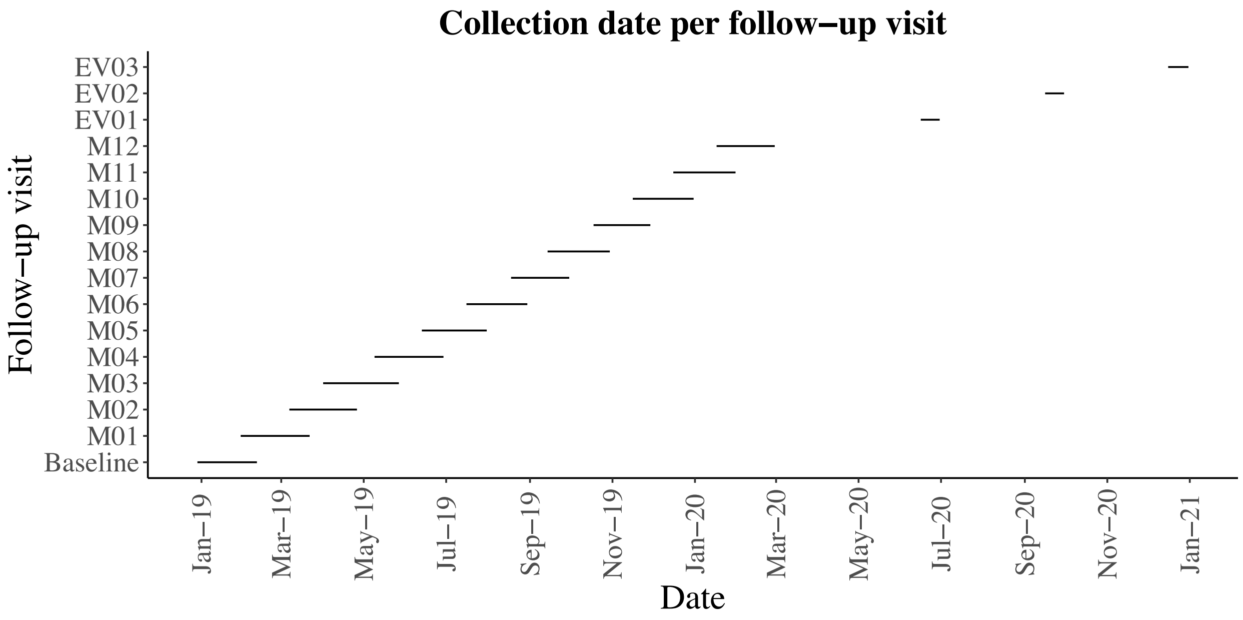

Figure S 1 *Timespan of visits per follow-up month.*

2. Data preparation and variable description

The demographic risk factors that were used in the statistical analyses are sex, age, and forest coverage of the subject's village of residence. Sex is a binary variable, taking values "male" and "female". Age was available as continuous data, but was recoded as a binary variable, taking the values "≤ 16 years", and "> 16 years", hereafter also called "children" and "adults". Forest coverage is a categorical variable taking the values "Inside forest", "Forest fringe", and "Outside forest". Residents of villages with ≥ 50% of households with ≥ 10% forest cover in their vicinity as of 2018 were considered living "inside the forest", with ≥30% of households with ≥5% forest cover as living "at the forest fringe" and living "outside the forest" otherwise. The definition of these risk factor variables was extracted from (Sandfort, et al. 2020).

In the statistical models including time, a linear time trend was included, called "Time (standardized)". This variable T was transformed from the continuous variable t ranging from 1, for the first day of the study, to 428 for the last day of the study, by centering around the mean and standardization:

$$T = \frac{t - \bar{t}}{SD(t)}$$

This transformation was performed for better readability of the time effect estimates.

3. Dominance of *P. vivax* over other *Plasmodium* species

**Species**	**Positive samples**	**% of samples**
P. vivax	879	8.85%
P. falciparum	25	0.25%
P. malariae	5	0.05%
P. vivax and *P. falciparum*	14	0.14%
P. vivax and *P. malariae*	1	0.01%
**Total infections**	**1010**	**10.17%**

Table S 1 *PCR-positive samples over the study period by species*

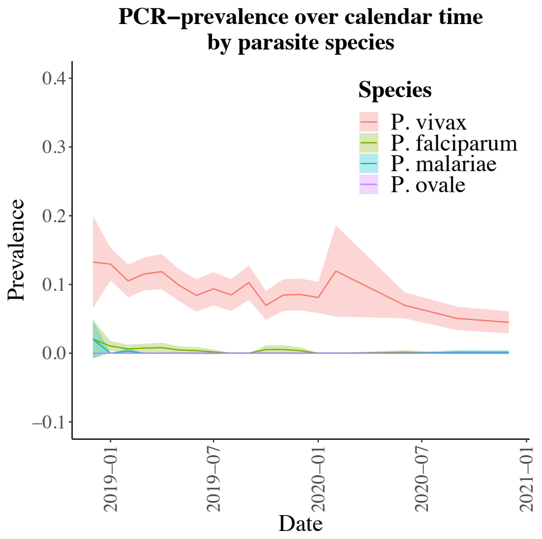

46

47 ***Figure S 2*** *Time-trends of* Plasmodium spp. *PCR prevalence (lines) with 95% binomial confidence interval (shaded area) by*
48 *parasite species.*

49 ## 4. Time-trends of malaria prevalence stratified by major demographic factors

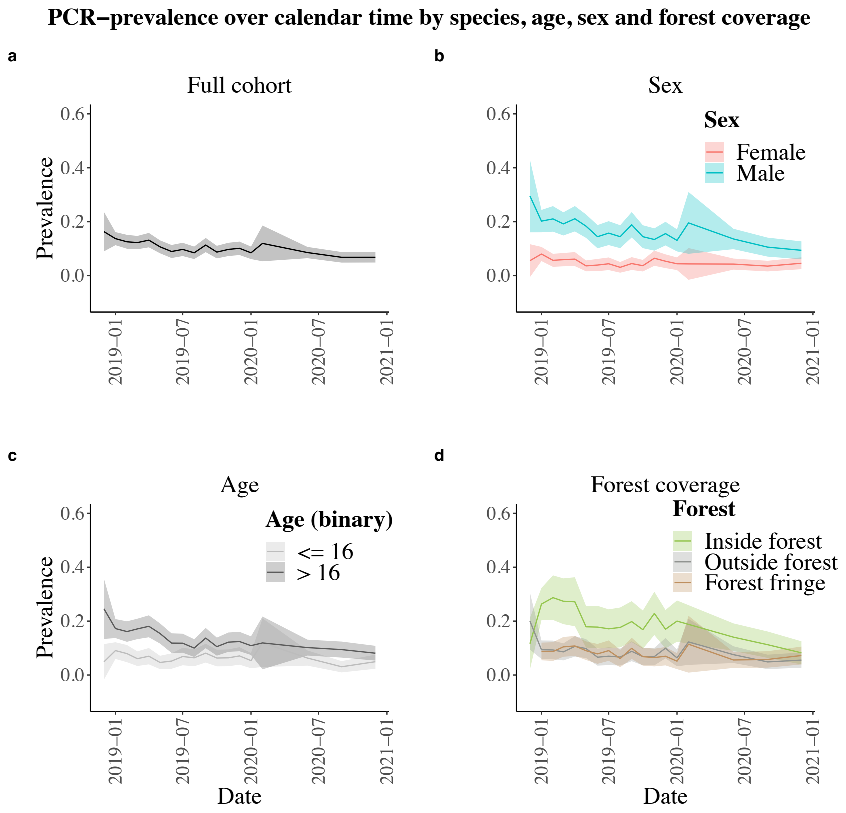

Figure S 3 *Time trends of* Plasmodium spp. *PCR-prevalence (lines) with 95% binomial confidence interval (shaded area)* ***a.*** *in the full cohort,* ***b.*** *by sex,* ***c.*** *by age group and* ***d.*** *by forest coverage.*

5. Risk factors for *P. vivax* PCR-positivity

a. Interaction of time and demographic risk factors

i. All demographic risk factors and two- and three-way interactions

	Estimate	Std. Error	p-value
(Intercept)	-8.051	0.846	0.000
Time (standardized)	-0.564	0.100	0.000
Sex (Male)	1.390	0.945	0.141
Age (> 16)	-0.279	1.081	0.796
Forest (inside forest)	3.957	0.980	0.000
Forest (forest fringe)	0.872	1.042	0.403
Sex (Male):Age (> 16)	3.873	1.253	0.002
Age (> 16):Forest (inside forest)	0.098	1.291	0.940
Age (> 16):Forest (forest fringe)	-0.827	1.447	0.568
Sex (Male):Forest (inside forest)	-1.289	1.260	0.307
Sex (Male):Forest (forest fringe)	-0.060	1.241	0.962
Sex (Male):Age (> 16):Forest (inside forest)	-1.644	1.655	0.321
Sex (Male):Age (> 16):Forest (forest fringe)	-0.236	1.702	0.890
Individual random intercept		3.226	

Table S 2 *Parameter estimates for a mixed-effects logistic regression of P. vivax PCR-infections against demographic variables and all two-and three way interactions.*

ii. Interaction with Sex

	Estimate	Std. Error	p-value
(Intercept)	-10.071	0.641	0.000
Time (standardized)	-0.613	0.204	0.003
Sex (Male)	3.183	0.429	0.000
Age (> 16)	1.834	0.387	0.000
Forest (inside forest)	3.071	0.469	0.000
Forest (forest fringe)	0.092	0.434	0.832
Time (standardized):Sex (Male)	0.123	0.235	0.602
Individual random intercept		4.411	

Table S 3 *Mixed-effects logistic regression of P. vivax PCR-infections against demographic variables and a time-sex interaction term.*

iii. Interaction with Age

	Estimate	Std. Error	p-value
(Intercept)	-9.876	0.634	0.000
Time (standardized)	-0.288	0.179	0.107
Sex (Male)	3.109	0.400	0.000
Age (> 16)	1.618	0.411	0.000
Forest (inside forest)	3.082	0.469	0.000
Forest (forest fringe)	0.094	0.435	0.829
Time (standardized):Age (> 16)	-0.344	0.218	0.115
Individual random intercept		4.414	

Table S 4 *Mixed-effects logistic regression of P. vivax PCR-infections against demographic variables and a time-age interaction term.*

iv. Interaction with Forest coverage

	Estimate	Std. Error	p-value
(Intercept)	-9.918	0.631	0.000
Time (standardized)	-0.383	0.169	0.024
Sex (Male)	3.101	0.400	0.000
Age (> 16)	1.802	0.388	0.000
Forest (inside forest)	2.850	0.500	0.000
Forest (forest fringe)	0.025	0.461	0.957
Time (standardized):Forest (inside forest)	-0.316	0.244	0.194
Time (standardized):Forest (forest fringe)	-0.096	0.254	0.704
Individual random intercept		4.433	

Table S 5 *Mixed-effects logistic regression of P. vivax PCR-infections against demographic variables and a time-forest interaction term.*

6. Risk factors for *P. vivax* PCR-positivity at baseline

	Estimate	Std. Error	p-value
(Intercept)	-3.115	0.344	0.000
Sex (Male)	0.648	0.384	0.092
Age (> 16)	0.018	0.383	0.963
Forest (inside forest)	1.200	0.246	0.000
Forest (forest fringe)	-0.320	0.278	0.250
Sex (Male):Age (> 16)	1.280	0.475	0.007

Table S 6 *Parameter estimates for P. vivax PCR-positivity at baseline. The reference level is a female child outside the forest.*

7. Risk factors for *P. vivax* seropositivity at baseline

a. *All demographic risk factors and two- and three-way interactions*

	Estimate	Std. Error	p-value
(Intercept)	-2.398	0.426	0.000
Sex (Male)	0.465	0.535	0.384
Age (> 16)	0.191	0.524	0.715
Forest (inside forest)	1.676	0.519	0.001
Forest (forest fringe)	0.318	0.585	0.586
Sex (Male):Age (> 16)	2.111	0.650	0.001
Sex (Male):Forest (inside forest)	-0.129	0.687	0.852
Sex (Male):Forest (forest fringe)	-0.192	0.743	0.796
Age (> 16):Forest (inside forest)	0.791	0.640	0.216
Age (> 16):Forest (forest fringe)	0.635	0.699	0.363
Sex (Male):Age (> 16):Forest (inside forest)	-1.392	0.879	0.113
Sex (Male):Age (> 16):Forest (forest fringe)	-0.958	0.890	0.282

Table S 7 *Parameter estimates for a logistic regression of P. vivax seropositivity against demographic variables and all two-and three way interactions.*

8. Survival analysis of *P. vivax* infection-free survival time

Month	Seropositive	Seronegative	Total
Baseline	293	641	934
M01	254	549	803
M02	183	505	688
M03	138	472	610
M04	119	445	564
M05	107	410	517
M06	100	391	491
M07	93	378	471
M08	91	369	460
M09	87	364	451
M10	82	358	440
M11	79	348	427
M12	74	340	414

Table S 8 *Number of individuals at risk of a PCR-infection at each follow-up month stratified by seropositivity status.*

**Inside forest**	χ^2	DF	p-value
Sex	0.028	1.000	0.867
Age	0.976	1.000	0.323
Seropositive	1.008	1.000	0.315
Sex:Age	0.063	1.000	0.802
Global	1.768	4.000	0.778

**Forest fringe**	χ^2	DF	p-value
Sex	0.082	1.000	0.775
Age	1.234	1.000	0.267
Seropositive	1.473	1.000	0.225
Sex:Age	1.162	1.000	0.281
Global	2.960	4.000	0.565

**Outside forest**	χ^2	DF	p-value
Sex	4.647	1.000	0.031
Age	2.733	1.000	0.098
Seropositive	4.086	1.000	0.043
Sex:Age	4.140	1.000	0.042
Global	7.039	4.000	0.134

Table S 9 *Proportional hazards test for models in* ***Table 4****.*

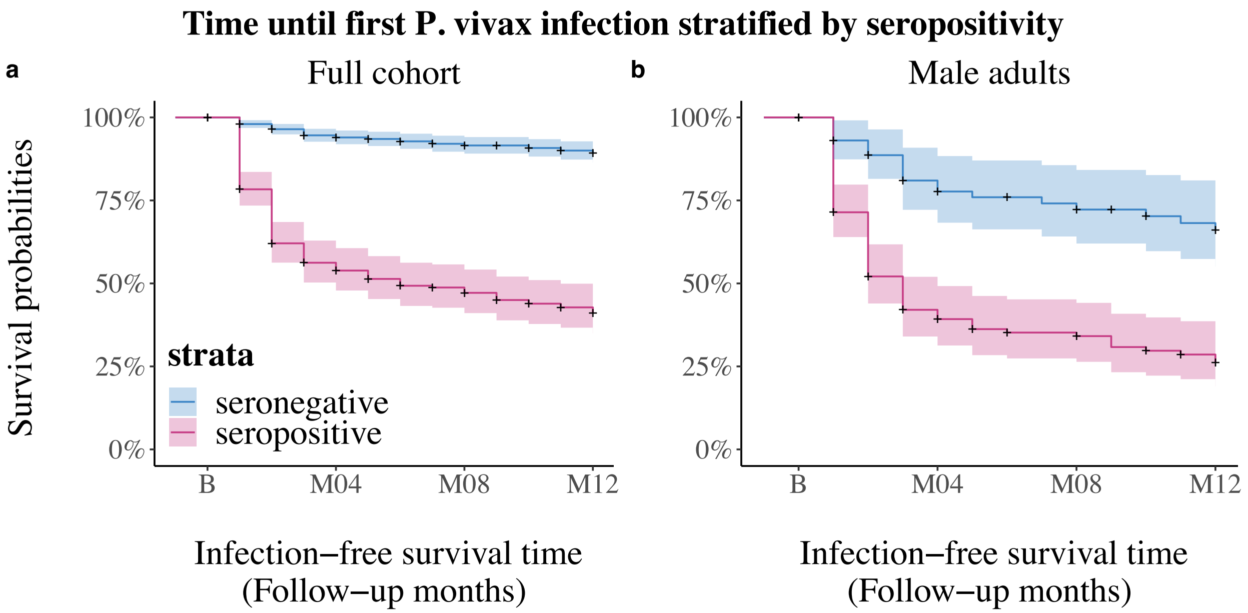

Figure S 2 *Kaplan-Meyer estimates of infection-free survival (lines) with 95% confidence interval (shaded area) stratified by seropositivity status at the cross-section for* ***a.*** *the whole cohort,* ***b.*** *male adults.*

Within adult males, seropositivity status at baseline was a significant predictor of infection-free survival over the study period. In male adults, we observed around 66.1% (55.1% - 79.4%) reinfection after the end of the study period for individuals who were seropositive at baseline, compared to only 26.2% (18.9% - 36.2%) reinfection in seronegative male adults.

	Estimate	Std. Error	p-value
Sex (Male)	0.448	0.450	0.319
Forest (inside forest)	1.096	0.327	0.001
Forest (forest fringe)	0.282	0.314	0.369
Age (> 16)	-0.377	0.477	0.429
Seropositive (yes)	0.495	0.282	0.080
Sex (Male):Age (> 16)	1.566	0.579	0.007

Table S 10 *Parameter estimated for a Cox-proportional hazards model of P. vivax infection including the full cohort.*
